# Ecosystem service provision in a changing Europe: adapting to the impacts of combined climate and socio-economic change

**DOI:** 10.1007/s10980-014-0148-2

**Published:** 2015-01-15

**Authors:** Robert W. Dunford, Alison C. Smith, Paula A. Harrison, Diana Hanganu

**Affiliations:** 1 Environmental Change Institute (ECI), Oxford University Centre for the Environment, South Parks Road, Oxford, OX1 3QY UK; 2 TIAMASG Foundation, Sfintii Voievozi 6, 010963 Bucharest, Romania

**Keywords:** Ecosystem services, Climate change impacts, Integrated assessment, Cross-sectoral interactions, Adaptation, Trade-offs

## Abstract

**Context:**

Future patterns of European ecosystem services provision are likely to vary significantly as a result of climatic and socio-economic change and the implementation of adaptation strategies. However, there is little research in mapping future ecosystem services and no integrated assessment approach to map the combined impacts of these drivers.

**Objective:**

Map changing patterns in ecosystem services for different European futures and (a) identify the role of driving forces; (b) explore the potential influence of different adaptation options.

**Methods:**

The CLIMSAVE integrated assessment platform is used to map spatial patterns in services (food, water and timber provision, atmospheric regulation, biodiversity existence/bequest, landscape experience and land use diversity) for a number of combined climatic and socio-economic scenarios. Eight adaptation strategies are explored within each scenario.

**Results:**

Future service provision (particularly water provision) will be significantly impacted by climate change. Socio-economic changes shift patterns of service provision: more dystopian societies focus on food provision at the expense of other services. Adaptation options offer significant opportunities, but may necessitate trade-offs between services, particularly between agriculture- and forestry-related services. Unavoidable trade-offs between regions (particularly South–North) are also identified in some scenarios.

**Conclusions:**

Coordinating adaptation across regions and sectors will be essential to ensure that all needs are met: a factor that will become increasingly pressing under dystopian futures where inter-regional cooperation breaks down. Integrated assessment enables exploration of interactions and trade-offs between ecosystem services, highlighting the importance of taking account of complex cross-sectoral interactions under different future scenarios of planning adaptation responses.

## Introduction

Climate change impacts on different sectors in Europe have been studied extensively (Kovats et al. 2014). However, fewer studies have examined the effects of climate change on ecosystem services. A review undertaken for the Intergovernmental Panel on Climate Change (IPCC)’s Fifth Assessment Report, by co-authors of this paper, identified 26 studies which reported findings on the potential impacts of climate change on ecosystem services in sub-regions of Europe based on an assessment of the published literature from 2004 to 2013 (Kovats et al. 2014). The review highlighted the following general trends: (i) all areas will experience loss in terms of at least one ecosystem service; (ii) the south will see losses across all three categories of provisioning, regulating and cultural services; (iii) provisioning services will increase in the north; (iv) regulating services will show both gains and losses in all regions (except the south); and (v) cultural services are expected to decline in the Continental, Northern and Southern regions, and show mixed trends in other regions.

Despite the implications of these changes for society and the environment, few studies have analysed how Europe might adapt to potential future climate change impacts on ecosystem services. To do this, it is essential that adaptation strategies are assessed within a range of potential socio-economic futures, as climate change impacts will interact with those associated with continuing social, economic and political changes, in potentially complex, non-additive ways (Harrison et al. 2014a). Furthermore, adaptation strategies will need to consider trade-offs between ecosystem services in order to assess which bundles of services can be delivered together (Raudsepp-Hearne et al. 2010) under varying and uncertain futures. Ignoring such trade-offs or cross-sectoral interactions can lead to either over- or under-estimation of climate change impacts and the need for adaptation (Holman et al. 2014).

This study attempts to address these knowledge gaps by modelling potential changes in the supply of ecosystem services across the different regions of Europe for four future climate and socio-economic scenarios, taking account of interactions between sectors. In addition, the potential of different adaptation strategies to mitigate any decline in future service provision is explored. Trade-offs between different services associated with the different adaptation strategies are discussed.

## Method

### The CLIMSAVE integrated assessment platform (IAP)

**Fig. 1:**
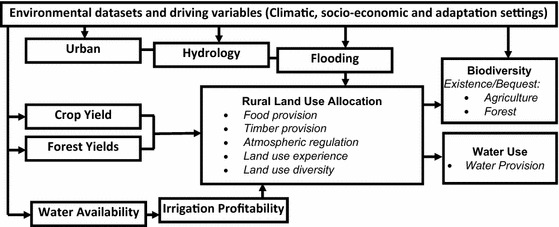
Simplified schematic showing the structure of the linked models within the European CLIMSAVE IA Platform. *Bullet points* in *italics* are the ecosystem services indicators used in this paper

The CLIMSAVE integrated assessment platform (IAP) was used to explore the impacts of climate and socio-economic change on ecosystem services. The IAP is an interactive, web-based, cross-sectoral modelling platform that includes interlinked meta-models for a number of sectors including urban development, agriculture, forestry, water provision, flooding and biodiversity (Harrison et al. 2013, 2014b). It draws climatic and socio-economic variables for any given user-selected scenario from a database and passes these to a chain of meta-models (Fig. 1) which determine the sectoral and ecosystem service outputs. The meta-models interact with one another: urban growth and flooding limit the areas available for other land uses; crop yield and forestry modules determine the influence of climate on the profitability of crops and trees, whilst the water availability module balances the supply and demand for water from different sectors to inform the profitability of irrigation. At the core of the network is the SFARMOD land allocation module (Audsley et al. 2014) which uses the relative profitability of crops and trees to determine the most profitable land use for all areas other than those areas protected for conservation. The biodiversity module at the end of the chain then uses the land use and information on water available for the environment to identify areas that have both suitable climate and habitat for a range of species. Detailed technical information about the meta-models and the original models on which they are based can be found in Harrison et al. (2013). The IAP results are presented at a 10′ by 10′ grid-cell resolution for the European Union, Norway and Switzerland. Baseline simulations using the IAP, representing the current situation, are based on the average 1961–1990 period for climate variables, such as temperature and precipitation, and 2010 for socio-economic variables, such as population and gross domestic product (GDP).

The IAP is an exploratory tool designed to help users better understand the complexities of interactions between multiple sectors in both different scenarios (both climate and socio-economic) and at a European scale. It is not intended to provide detailed local predictions or inform local planning, but to assist users in developing their capacity to address regional/national/European-scale issues surrounding climate change. The Platform also provides an educational component; it can be used as a teaching tool to help current students (potential future decision-makers) better understand the complexities surrounding adaption in Europe.

### Ecosystem service indicators

Eight ecosystem service indicators were selected from the outputs available from the IAP to cover a broad range across the three service categories: (i) provisioning services, that provide goods from ecosystems, such as food, timber and water; (ii) regulating services, that mediate environmental systems, such as climate regulation via carbon sequestration; and (iii) cultural services, that are the non-material benefits humankind derives from ecosystems, such as experiential interactions with the landscape and recreation.

Ecosystem service indicators for food, water and timber provision as well as atmospheric regulation in terms of carbon storage are all direct outputs from the IAP. For food provision, the indicator is *food production* in 1,000 s of KCal capita^−1^ day^−1^; this can be compared with the recommended daily allowance (for males) of 2,500 kcal. For water provision, the *Water Exploitation Index (WEI)* is used. This is the proportion of water availability that is required by water demand for domestic, power, industrial and agricultural purposes: i.e. 1-WEI is the amount remaining to maintain ecological flows (Wimmer et al. 2014). River basins with WEI below 0.2 are classified as “low” water stress and those above 0.4 as “severe” water stress (Alcamo et al. 2007). Timber provision is calculated as the total *annual forest yield from managed forests* (Mt year^−1^). Atmospheric regulation is represented by the *total carbon stored in the biomass of areas under intensive agriculture, extensive agriculture, forests and unmanaged land* (Mt year^−1^).

No indicators for cultural services are directly output from the IAP. Proxies for four cultural services have been developed from the IAP outputs. The *Landscape Experience* index, is designed to reflect the physical and experiential interactions with the landscape; its overall “naturalness”. This index is calculated as the total proportion of land uses that are not managed intensively for provisioning services, i.e. unmanaged land, unmanaged forest, extensive grasslands and set-aside. *Land Use Diversity* addresses a different aspect of land-use by focussing on the multi-functionality of the landscape. It is an indicator of balance; high values reflect multi-functional landscapes which have the potential to supply a broader range of different ecosystem services. It is calculated at the grid-cell level as the Shannon index of six major land use classes (forestry, arable, intensive grassland, extensive grassland, abandoned land and urban) and then averaged for broader regions.

“*Forest*” and “*Arable” Biodiversity Existence/Bequest* indices were also calculated for species associated with forest and arable habitats. Within the IAP, the SPECIES bioclimatic envelope model identifies, for selected species, where potential future climate-space and habitat may overlap (climate-habitat space) and compares this with the climate-habitat space at baseline. Following Berry et al. (2006), abiodiversity index is calculated for each species based on: (i) the amount of climate-habitat space that remains stable; (ii) the amount of existing climate-habitat-space that is lost; and (iii) the proportional coverage of the region in question with suitable climate-habitat space. Gains in climate-habitat space are not included in the index; it represents a worse-case scenario where species are unable to migrate to these areas. Six arable species and ten forest species are modelled in the IAP. These species are, for arable: Common poppy (*Papaver rhoeas*); Brown hare (*Lepus europaeus*); Linnet (*Cardueliscannabina*); Grey partridge (*Perdix perdix*); Pheasant (*Phasianus colchicus*); Rabbit (*Oryctolagus cuniculus*)and for forest: Hornbeam (*Carpinus betulus*); Bilberry (*Vaccinium myrtillus*); Norway spruce (*Piceaabies*); Brown bear (*Ursusarctosarctos*); Cowberry (*Vaccinium vitis*-*idaea*); Roe deer (*Caproeluscapreolus*); Lynx (*Lynx lynx*); Purple emperor butterfly (*Apatura iris*); Wild boar (*Sus scrofa*); Woodcock (*Scolopax rusticola*). Results for these species were averaged to create two separate aggregate indices which were then inverted and standardised to a value between zero (no appropriate climate-habitat space remains) and one (100 % stable climate-space and geographical coverage >50 % of the region); the baseline value for the indicator is then subtracted from the scenario value to create a change from baseline indicator: as such it is not possible to interpret either index at baseline.

### Scenarios

To represent the range of potential future climates, the IAP contains data for five Global Climate Models (GCMs: CSMK3, MPEH5, HadGEM, GFCM21, IPCM4), four IPCC SRES emissions scenarios (A1b, A2, B1 or B2) and three levels of climate sensitivity (low, medium or high). Pattern scaling is used to combine these data into climate scenarios (Dubrovsky et al. 2014). Any climate scenario can be run for either baseline conditions, the 2020s or 2050s and combined with one of four socio-economic scenarios developed at a series of international stakeholder workshops (involving individuals from government, NGOs, the private sector, research and media; Gramberger et al. 2014). These socio-economic scenarios present four futures located at the extremes of two axes of “economic development” and “innovation success” (Kok et al. 2014); they are designed to test the extent to which approaches to adaptation are robust to divergent socio-economic conditions. For this study, a subset of the scenarios for the 2050s from the IAP was used and the influence of adaptation options within these combined climate and socio-economic scenarios were explored. The scenarios included: (i) two climate scenarios (one moderate, one extreme); (ii) two socio-economic scenarios (one utopian, one dystopian); and (iii) eight adaptation strategies each targeting particular ecosystem services.

#### Climate scenarios

The “extreme” climate scenario is based on the GFCM21 model with an A1 emissions scenario and high climate sensitivity; this scenario shows area-average temperature increases of just over 3 °C for Europe and strong precipitation decreases of around 30 % in summer (it is the driest of all five GCMs). It has a spatial pattern that shows changes in precipitation ranging from −42 % in the south of Spain and Italy to a maximum increase of +24 % in areas of Fennoscandia; mean temperature increases in a southerly and easterly direction from ≈+1 °C in the northern UK to >3 °C warming in much of southern Europe and northern Finland.

The “moderate” climate scenario is based on the IPCM4 model with a B2 emissions scenario and low climate sensitivity; this scenario shows a north-west trend in increasing temperature with a maximum increase of 2.2 °C and changes in annual precipitation from −10.4 % in southern Europe to +7.9 % in north-west Europe. These scenarios were run as climate-only scenarios using baseline socio-economic conditions (so all changes result solely from differences in climate) and in paired combinations with the two socio-economic scenarios (Table 1).

**Table 1:** Overview of the combined climate and socio-economic scenarios, and the adaptation strategies

	Scenario count	Climate setting	Socio-economic settings	Adaptation settings
Baseline	1	Baseline climate	Baseline socio-economics	None
Climate only	2	1× Extreme climate scenario 1× Moderate climate scenario	Baseline socio-economics	None
Combined climate and socio-economic scenarios	4	1× Extreme climate scenario & SoG socio-economic scenario 1× Extreme climate scenario & WRW socio-economic scenario 1× Moderate climate scenario & SoG socio-economic scenario 1× Moderate climate scenario & WRW socio-economic scenario		None
Adaptation strategies	4 × 8	8× adaptation strategies for each of the four combined climate and socio-economic scenarios (see Table 2)		8× strategies
Total	39			

#### Socio-economic scenarios

The utopian “We are the world” (WRW) and the dystopian “Should I stay or should I go” (SoG) scenarios were selected from the stakeholder-derived scenarios. In WRW (characterised by successful innovation and stable economic growth) there is an effective government, a focus on well-being and wealth redistribution, reduced inequality, more global cooperation and a conflict-free world. Population increases moderately (+5 %). Conversely, in SoG (characterised by unsuccessful innovation and rollercoaster economic decline) there is a failure to address economic crises, political instability, increased inequality and an insecure, unstable world. Population increases rapidly (+23 %). Both socio-economic scenarios have their own input settings, determined at the stakeholder workshops and by IAP experts, for a range of variables which lead to different levels of impact. For example, WRW, where innovation is successful, has more optimal values for “water saving due to technological change” and higher “crop yields” as a result of improvements in crop breeding and agronomy. Each socio-economic scenario was run in combination with each climate scenario in the absence of adaptation. The adaptation strategies below were then run for each combined climate and socio-economic scenario (Table 1).

#### Adaptation strategies

Adaptation is implemented within the IAP in terms of “slider” controls which the user can alter to change the socio-economic inputs to the modelling system. These include options such as: enlarging protected areas or improving crop yields or water savings through changes in technology or behaviour. The limits within which any of these adaptation options can be implemented change with the socio-economic scenario and are reflected by different maxima, minima and default values set by stakeholders and modellers during the scenario workshops. For example, it is possible to increase water savings through technological change much less in the dystopian scenario (where innovation has failed) than in the utopian scenario. Eight adaptation strategies were created to target different ecosystem services by combining different adaptation options (Table 2). This was implemented by setting the adaptation sliders to the most beneficial extreme value of the given socio-economic scenario (Table 2).

**Table 2:** Adaptation strategies as applied within each combination of climate and socio-economic scenario

Adaptation Strategies	Settings (↓ decrease to minimum ↑ increase to maximum)
1. Food self-sufficiency: Food imports are reduced to the minimum to encourage European food self-sufficiency	[*Food Imports*] ↓
2. Irrigation for food: This strategy is a combination of “food self-sufficiency” and “maximising water efficiency”. Water is prioritised for agricultural use	[*Food Imports*] ↓ [*Irrigation efficiency*] ↑ [*Water savings (technology)*] ↑ [*(behavioural)*] ↑ [*Water demand prioritisation*] = “prioritise food production”
3. Maximising water efficiency: Water provision is made a top priority. Adaptation approaches include more efficient irrigation and technological and behavioural changes	[*Irrigation efficiency*] ↑ [*Water savings (technology)*] ↑ [*Water savings (behavioural)*] ↑ [*Water demand prioritisation*] = “baseline”
4. Extensify agriculture: This strategy aims to reduce the impact of intensive farming on the environment by farming less intensively (which reduces yield) and putting more of a field into set-aside	[*Change in yields*] ↓ [*Set*-*aside*] ↑
5. Dietary change: Strategy based on “extensify agriculture” but with reduced pressure on food resulting from reduced dietary preferences for land-intensive red and white meat	As “extensify agriculture” plus: [*Change in diet (lamb/beef)*] ↓ [*Change in diet (chicken/pork)*] ↓
6. Maximising timber: This strategy focuses on timber production by planting species that best match the future climate and reducing agricultural demand by increasing imports	[*Food Imports*] ↑ [*Tree species*] = “Optimum” (all regions)
7. Forests for nature: Strategy based on “maximise timber” with additional forestry protected to increase the amount of total forest	As “maximise timber” plus: [*Protected Area Change*] ↑ [*Protected Area that is Forest*] = 100 % [*Method for Protected Area allocation*] = “Buffering then connectivity”
8. “Go nature go!”: Target overall naturalness: forest, extensive grassland, unmanaged land. Expand protected areas (PA) to equally target these land uses; deliberately target new areas rather than buffering existing PA. Plant competitive tree species; import as much food as possible; increase food yields and change dietary preferences to minimise agricultural pressures	[*Food Imports*] ↑ [*Protected Area Change*] ↑ [*PA Forest*] and [*PA Agriculture*] = 33 % [*Method for PA allocation*] = “Connectivity then buffering” [*Tree species*] = “Optimum” (all regions) [*Food yields*] ↑ [*Change in diet (lamb/beef)*] and [*Change in diet (chicken/pork)*] ↓

The strategies are created by modifying IAP slider settings to the maximum/minimum scenario-consistent settings as set out in the settings column above

**Fig. 2:**
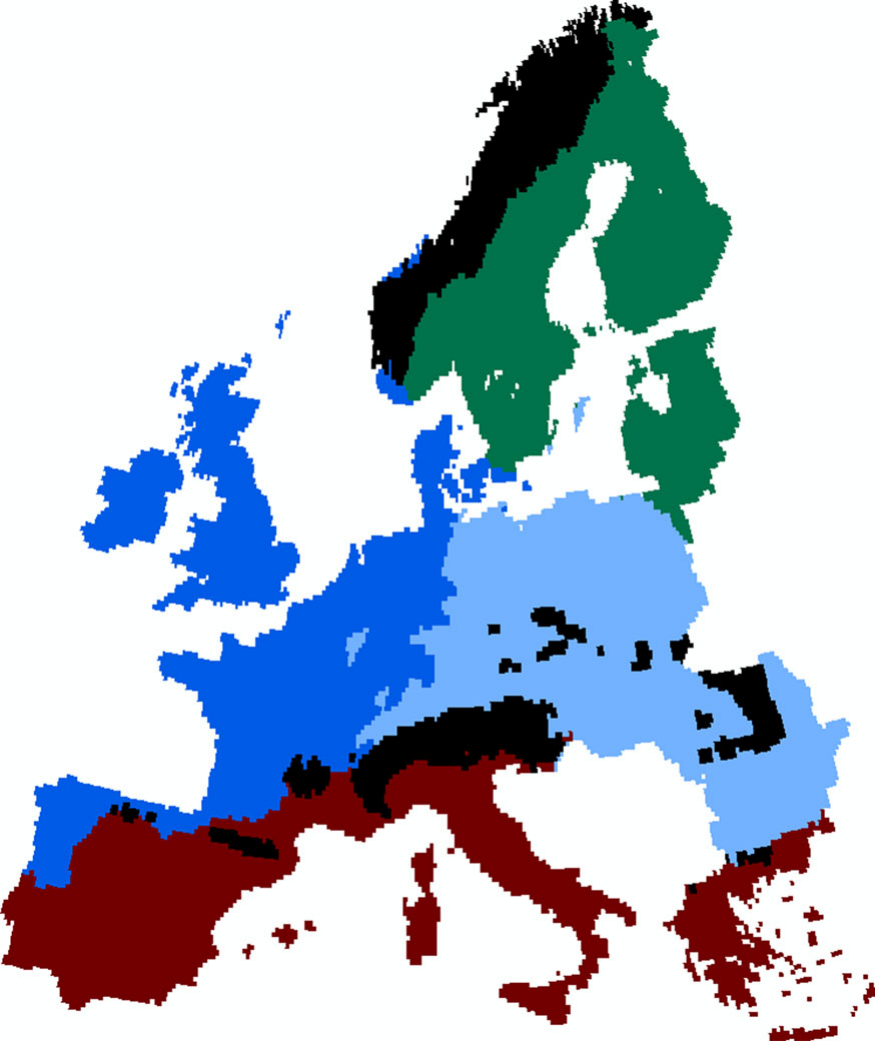
The regions considered within this analysis based on those defined by Metzger et al. (2005) and used within the IPCC AR5 Europe chapter (Kovats et al. 2014)

In total, 39 scenarios were run (Table 1) and the eight ecosystem service indicators were calculated for each. This data was then summarised for six regions: Europe, and the five European regions defined by Metzger et al. (2005) and used within the IPCC AR5 Europe chapter (Kovats et al. 2014) (Continental, Alpine, Atlantic, Northern, Southern; see Fig. 2).

### Modelling considerations

As with any modelling approach, the results must be understood in the context of the limitations of modelling framework in question. The IAP is intended as an exploratory tool for investigating alternative scenarios, rather than as a predictive tool to estimate absolute values for ecosystem service provision. As such, the focus should be on the general trends in services and the comparison between different scenarios.

There are a number of fundamental assumptions within the meta-models that need to be considered when interpreting the results. Firstly, the land use allocation meta-model is driven by demand for food production (grain, meat, etc.) as determined by scenario parameters such as dietary preference and the amount of these commodities imported (Audsley et al. 2014). The meta-model iterates to meet this demand, meaning that food provision is prioritised within the system. Whilst it is realistic to assume that Europe would ensure that it was able to meet its food demand, this approach means that the modelling involves *autonomous* adaptation in the food sector. However, this demand is met in very different ways in different climate and socio-economic scenario combinations, as well as under the different adaptation strategies. Furthermore, in scenarios where food provision is not a priority, considerable pressure can be placed on the food sector, particularly in areas at a regional scale with low food provisioning potential, such as the Alps. The land use modelling is also based on the fundamental assumption that the most profitable use will be made of the land, unless that land is in a protected area. In reality, land use change may not be driven entirely by profit, but also by historical and cultural factors, so this assumption should be taken into consideration when interpreting the results.

The biodiversity indices also need to be carefully interpreted. Arable and forest species were selected to explore the impacts of climate and habitat change, and habitat was assumed to be lost if arable or forest area was removed from a pixel where it had previously overlapped with the species occurrence. However, some of the species may be able to make use of alternative habitats (such as extensive grassland or unmanaged land). Although the extent to which this is possible will vary with the species in question, the index should be interpreted with this in mind. It should also be noted that the biodiversity indices do not take account of differences in land management that can have significant impacts on biodiversity, such as the distinctions between different farming techniques, or between managed and unmanaged forest.

## Results

### Baseline ecosystem service distribution

At baseline and at a European scale, food production is adequate, five times an adult male’s recommended allowance; water provision stress is “low”, the proportion of water used is 12 % of the water available; 262 Mt of timber are produced a year; 7,453 Mt carbon is sequestered annually as biomass; 32 % of land use is not dedicated to provisioning services; and the Shannon index of diversity is moderate (0.5; Table 3).

**Table 3:** Baseline ecosystem service distribution across the European regions

	Food provision food production (1,000 s of KCal capita^−1^ day^−1^)	Water provision water exploitation index (no units)	Timber provision annual forest yield from managed forests (Mt)	Atmospheric regulation carbon sequestration (Mt/year)	Landscape experience land not managed for provisioning services (%)	Land use diversity Shannon index of land use
EU	13.2	0.12	262	7,453	32	0.50
Northern	22.1	0.03	80	2,020	37	0.42
Alpine	7.9	0.04	35	1,112	65	0.35
Atlantic	12.3	0.16	70	1,589	15	0.51
Continental	13.5	0.19	45.5	1,914	16	0.58
Southern	13.3	0.22	32	818	44	0.60

The biodiversity index is a change from baseline index and as such has no value at baseline

However, there are significant inter-regional variations. At baseline, the Northern region has an advantage in terms of the supply of many ecosystem services. Dominated by managed forestry, with a low population but more arable crops and intensive grassland than the Alpine region, it performs best in terms of food, water and timber provision, atmospheric regulation and forest biodiversity. However, the domination of managed forestry means that the north scores less well than the Alpine region for both landscape experience (with 37 % non-provisioning rather than 65 %) and land use diversity (managed forestry makes up 44 % of land use). Conversely, the Southern region is the only region under “moderate” water stress even at baseline and produces less biomass carbon and timber than the other regions. The Alpine region produces the least food of all the regions: only three times adult male requirements (7,900 kcal day^−1^), compared to ≈5 times in most other regions and ≈9 times in the sparsely populated Northern region (22,100 kcal day^−1^). In terms of land use, the Alpine region, which is largely dominated by unmanaged land (46 %), has the lowest land use diversity but the greatest value for the land use experience index (with 67 % of land not being managed for provisioning services). The Atlantic and Continental regions fall between these two extremes producing significant amounts of food, but with large populations leading to food provision per capita results close to the European average (≈5 times recommended allowance).Both regions are also close to the border between low and moderate water stress (WEI = 0.19/0.16 for Continental/Atlantic regions, respectively). The Atlantic region produces more timber than the Continental region (26 % of the European total, just less than the Northern region’s 30 %) whilst the Continental region sequesters more carbon as biomass (25 % of European total, just less than the Northern region’s 27 %). Both regions have very high proportions of land dedicated to provisioning services (with only 15–17 % left to other uses) and so score lowest for landscape experience. However, with a mix between arable, intensive grassland and managed forestry they score well for land use diversity.

### The impact of climate change on ecosystem service provision (the climate-only scenarios)

**Fig. 3:**
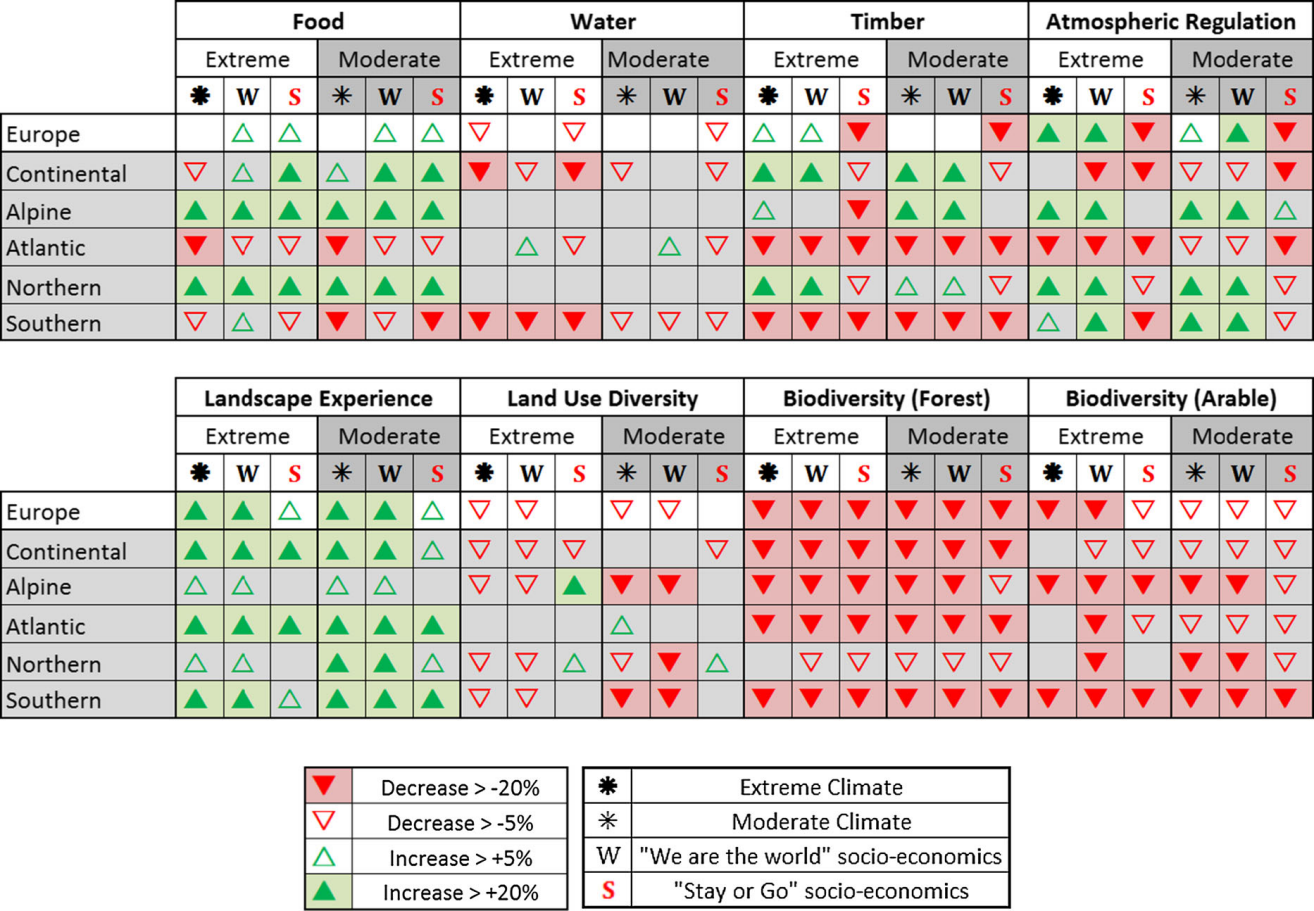
Impacts of climate change and socio-economic scenarios on sectors. Changes are relative to the European baseline climate (1961–1990)

At the European scale and in the absence of socio-economic change, all services, with the exception of food provision (both climates) and timber provision (moderate climate only), are projected to change by greater than ±5 % from their baseline values (Table 4; Fig. 3). Under the extreme climate, atmospheric regulation and landscape experience increase by >20 %, whilst stress on water provision increases by 20 % and the arable and forest biodiversity indicators decrease by ≈30 and 40 %, respectively. Under the moderate climate, changes are less severe, with only ‘landscape experience’ increasing by >20 %. These changes reflect not only the direct influence of climate change, but its knock-on impacts on land use allocation and land management. Unmanaged land nearly doubles in size, increasing from 16 to 29/33 % of the total area in the extreme/moderate scenarios, respectively. This change is at the expense of managed forest (which decreases by 8 % in total area under both climates), and total (intensive + extensive) grassland (−4 % extreme; −6 % moderate) and to a lesser extent arable land (−1 %; −2 %) as land use patterns shift to ensure food provision under the new climates. These shifts also reflect an increased intensity of use: as less area is dedicated to food provision (as reflected by the increase in land use experience) there is a greater dependence on management practices such as irrigation to ensure adequate food supply. Stress on water provision increases as a result of both a reduction in water availability (−14 % in the extreme scenario; −3 % in the moderate) and the water demand for agriculture (which increases by ≈200 % relative to baseline in the extreme scenario and 90 % in the moderate).

**Table 4:** Impacts of climate change and socio-economic scenarios on ecosystem services at the European scale

	Food provision food production (1,000 s of KCal capita^−1^ day^−1^)	Water provision water Exploitation Index (no units)	Timber provision annual forest yield from managed forests (Mt)	Atmospheric regulation carbon sequestration (Mt/year)	Landscape experience land not managed for provisioning services (%)	Land use diversity Shannon index of land use	Forest biodiversity existence/bequest biodiversity vulnerability index (no units)	Arable biodiversity existence/bequest biodiversity vulnerability index (no units)
Baseline (Europe, 1990)	13.2	0.12	262	7,453	34	0.50	0.00	0.00
Extreme climate (2050s)	13.0	0.17	294	9,246	46	0.47	−0.40	−0.29
Extreme climate & WRW	14.6	0.12	281	9,439	45	0.45	−0.40	−0.31
Extreme climate & SoG	15.2	0.21	167	5,150	40	0.51	−0.44	−0.16
Moderate climate (2050s)	12.9	0.14	269	8,719	46	0.46	−0.23	−0.17
Moderate climate & WRW	14.6	0.09	269	9,090	45	0.44	−0.23	−0.18
Moderate climate & SoG	15.2	0.17	198	5,679	39	0.51	−0.24	−0.09

### The impact of climate and socio-economic change on ecosystem service provision (the combined climate and socio-climatic scenarios)

#### Results for the European scale

**Fig. 4:**
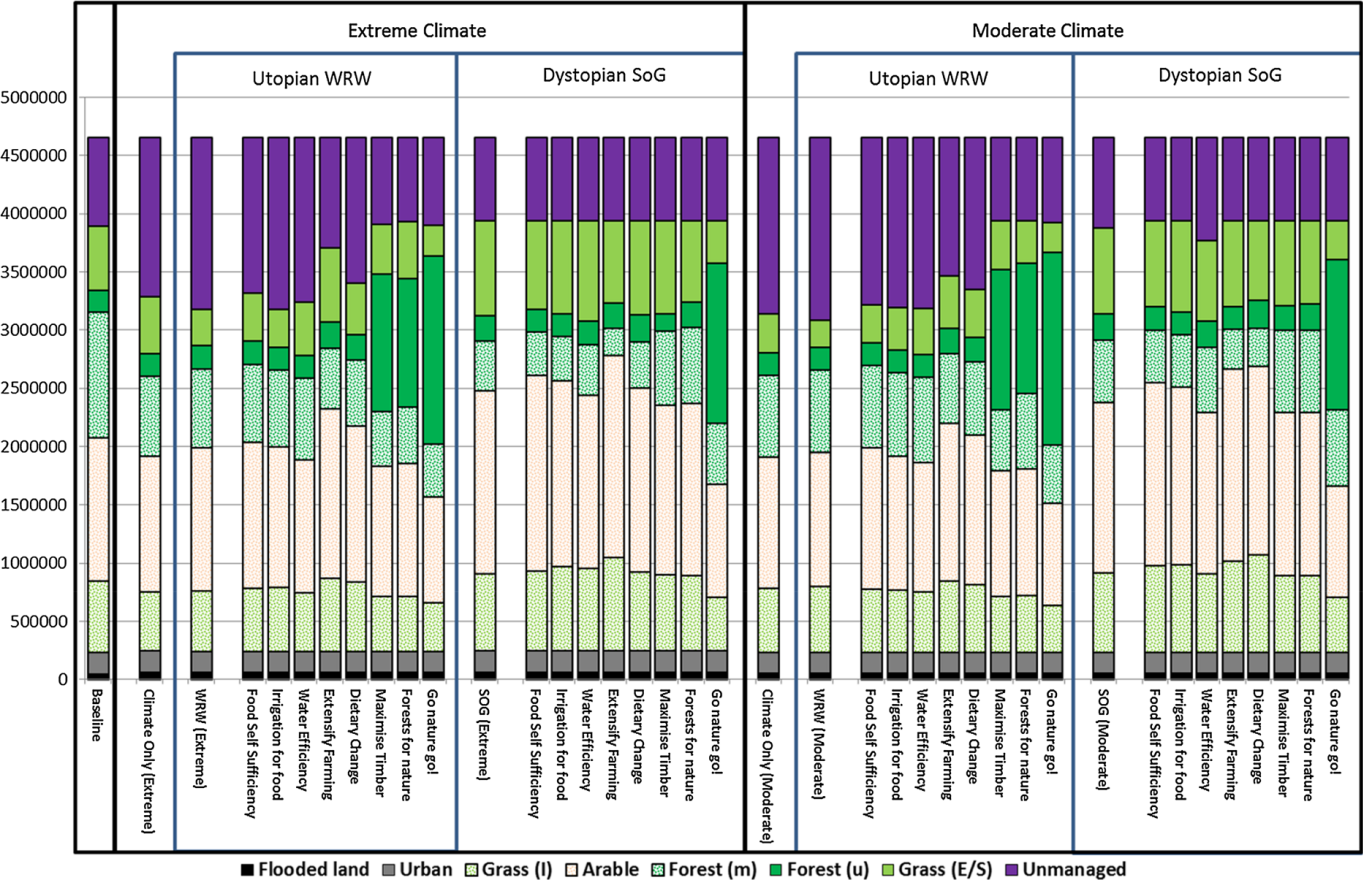
Changing land use with climate and socio-economic scenarios and adaptation strategies. Units are area (km^2^). Grass (I) and (E/S) are “intensive grassland” and “extensive grassland and set-aside” respectively; Forest (m) and (u) are managed and unmanaged forest

The impact of the combined climate and socio-economic scenarios on ecosystem services varies considerably from those of the climate-only scenarios. In the dystopian scenario (SoG), pressure from an increasing population and failed innovation means that considerable stress is placed on the agricultural system to feed the population. As such, food production for SoG increases by 17–18 % relative to the climate-only scenarios. However, failed innovation means that irrigation efficiency in SoG is 21 % lower than at baseline. As such, the use of irrigation is limited as a means to increase food provision in farming areas irrigated at baseline. Instead, the model computes that it is more cost-effective to expand agriculture, leading to significant land use change: intensive grassland, arable and extensive grassland increase (+3.4 %; 7.0 and 8.6 % total area, respectively), whilst forests and unmanaged land decrease (−5.0 and −14.0 % total area, respectively). As such, increases in food provision result in trade-offs with other ecosystem services, specifically a reduction in timber provision and atmospheric regulation (both >−20 % relative to the climate-only scenario; Fig. 4) with a knock-on effect of decreasing forest biodiversity (−7 % from climate-only). In the water sector, SoG’s move away from irrigation actually reduces agricultural demand for water under the moderate climate (−2 %; 0.9bn m^3^ less); however, this is not the case under the extreme climate where it increases by +10 % (13.7bn m^3^) compared to the climate-only simulation. Nevertheless, under both climates SoG’s failure in innovation in terms of water savings leads to the domestic and power sectors demanding considerably greater proportions of water (+23.2bn m^3^ from domestic and +35.6bn m^3^ for power). These changes mean that overall water demand increases by ≈25 % from baseline in both climates, leading to an increase in a WEI already stressed by climate. Under the extreme climate, this leads to the European WEI exceeding the 0.2 threshold indicating ‘moderate’ rather than ‘low’ water stress; a very significant change given the continental scale. Conversely, species which are dependent on arable habitats are less vulnerable in SoG than at baseline (or in WRW) as arable land use expands. Similarly, due to the agricultural expansion “land use diversity” increases as, at a grid-cell level, there are more cells with a wider mix of land uses under SoG than at baseline, or under the climate-only scenarios or the utopian scenario, WRW.

We are the world is a considerably different situation to SoG. Population growth, though moderate compared to SoG, leads to an increase in food demand and food provision increases to meet this demand (≈+12–13 %). However, in contrast to SoG, this demand is not met by broad-scale land use change: extensive grassland decreases (−36 %), replaced by abandoned land, but all other land use classes remain within 0–10 % of their values under the climate-only scenarios. This is possible because in WRW successful innovation means that irrigation is 26 % more effective and agricultural yields have increased by 15 % due to improvements in agronomy, meaning that more food can be produced in less space and without needing to move to access water. As a result there is considerably less change in ecosystem service provision, particularly in terms of timber provision, atmospheric regulation, landscape experience, land use diversity and both biodiversity indices where all changes are <±5 % compared with the same climate-only scenario. Furthermore, in WRW there are positive improvements in terms of water stress which *decreases* relative to the comparable climate-only scenario (−0.05 WEI units) due to improvements in water savings from technology and behaviour.

#### Results for European sub-regions

Compared with the European scale, the regions of Europe respond differently to both the climate and socio-economic scenarios (Fig. 4). Some regional trends are consistent across all climate and socio-economic scenario combinations: (i) food provision increases in the Northern and Alpine regions and declines in the Southern region, even in the climate-only scenarios where there is no overall trend in food provision at the European scale; (ii) stress on water provision increases in the Continental and Southern regions, particularly under the extreme climate– the stress in the Southern region reaches levels of “severe” water stress under an extreme climate (WEI = 0.93 in SOG; 0.52 in WRW) and “moderate” water stress even under the moderate climate; (iii) timber provision decreases in the Atlantic and Southern regions; (iv) atmospheric regulation decreases in the Atlantic region; (v) landscape experience increases in all regions, but less so in the Alpine and Northern regions which were relatively high at baseline; (vi) the biodiversity indicators decrease in the majority of regions and scenarios, however, forest species decrease less in the Northern regions and the arable species decrease less in the Continental and Atlantic regions, whilst the Alpine and Southern regions are vulnerable even in the climate-only scenarios.

Other regional trends show different directions dependant on the scenario. Food provision, for example, decreases in the Southern region in the climate-only and SoG scenarios, but shows an increase in food production in the WRW scenario where irrigation is more efficient. Similarly, the Continental and Northern regions show increases in terms of timber production in the climate-only and WRW scenarios, but reductions relative to baseline in the SoG scenario; the Northern and Southern regions show a similar trend in terms of atmospheric regulation.

The existence of some consistent regional trends across climate and socio-economic scenarios highlights key potential risks and opportunities in terms of ecosystem service changes in these regions. However, the existence of regional trends that differ between scenarios stresses the significance of socio-economic drivers in shaping future service provision and the importance of assessing different adaptation responses under a wide range of climate and socio-economic futures.

### The influence of adaptation in responding to the impacts of climate and socio-economic change on ecosystem services (the adaptation strategies)

#### Adaptation strategies focused on food and water provision

Three of the adaptation strategies focus primarily on food and water provision. “Food self-sufficiency” and “irrigation for food” both explore the role of adaptation in a future where Europe’s reliance on its own food production increases due to reductions in the amount of food imported (−19 %). However, “irrigation for food” also adds adaptation options that increase water savings in all sectors, including increasing irrigation efficiency, and prioritises water for food production over other uses. The third scenario, “water efficiency” complements these by focussing solely on the water sector, using the same water-saving adaptation options as “irrigation for food” but food imports are set to the scenario default (−13 %).

**Fig. 5:**
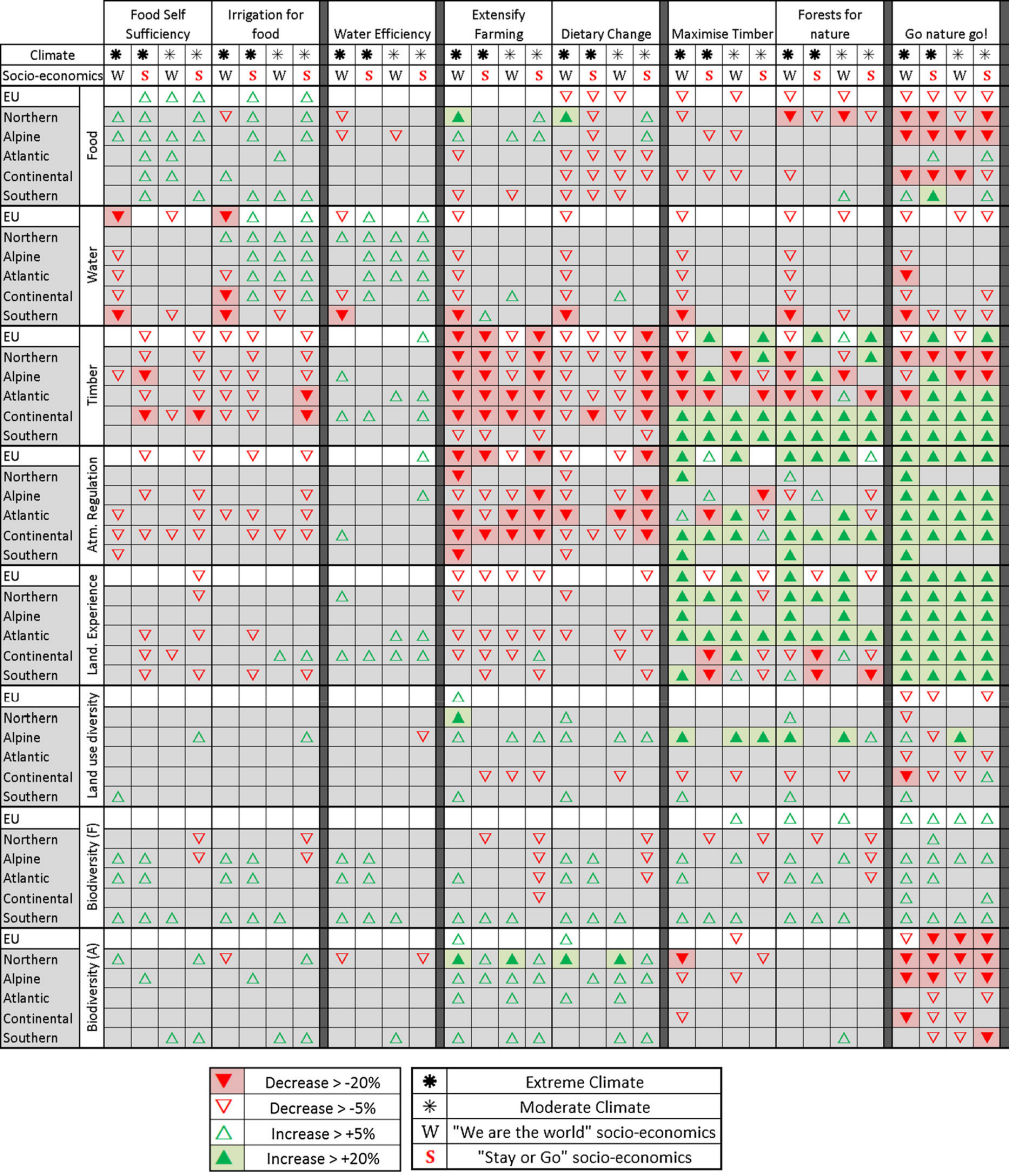
The influence of adaptation strategies on ecosystem services. Changes are relative to the combined climate and socio-economic scenario without adaptation

At the European scale the two low-import strategies increase the total amount of food produced (>+5 %) in at least one scenario and have a positive influence on regional food provision in multiple scenarios (Fig. 5). However, there are notable differences: “food self-sufficiency” shows an >5 % increase in food provision in three of the four combined climate and socio-economic scenarios at the European scale (compared to two for “irrigation for food”) and has the greatest impact at a regional scale, with 13 of the 20 (65 %) region/scenario combinations showing increases in food provision >+5 % (compared to 9 (45 %) for “irrigation for food”).

The spatial differences are also interesting. “Irrigation for food”, for example, has a very different influence on the spatial pattern of food provision in the WRW scenario under extreme climate conditions: it reduces food provision in the Northern region, and increases it in the Continental region. This suggests that in a utopian scenario, even under extreme climate pressure, improvements in water savings and improved irrigation enable enough production in other regions to actually reduce the need for agriculture in the north. In contrast, without these water savings (i.e. under the “food self-sufficiency” adaptation strategy) the model increases food production in both the Northern and Alpine regions. In terms of impacts on water provisioning, the “irrigation for food” strategy demonstrates that with improvements in technology it is possible to balance food and water provisioning in most regions and scenarios. The Northern, Alpine and Atlantic regions show improvements (+>5 %) in the majority of scenarios (Fig. 5).

However, at a regional scale, all three adaptation scenarios are shown to be capable of *increasing* WEI. For example in the extreme-climate WRW scenario the Continental WEI increases from 0.22 in the no adaptation scenario to 0.25, 0.26 and 0.30 with the “water efficiency”, “food self-sufficiency” and “irrigation for food” strategies, respectively. This increase reflects rebound effects that result from increased water savings making irrigation more cost-effective: areas that were not profitable to irrigate prior to adaptation are now farmed, with an overall result of less water being available for ecological purposes (thus higher WEI).

With regard to the other ecosystem services, both the low-import adaptation strategies have generally negative impacts on timber and atmospheric regulation, particularly under dystopian socio-economic conditions, in all regions but the south (Fig. 5). There is little influence on land use diversity beyond that already resulting from the combined climate and socio-economic scenarios. In terms of biodiversity, responses are generally positive relative to the scenarios without adaptation (loss of forestry is refocused to other regions) and arable biodiversity increases in the Northern or Atlantic regions depending on the socio-economic scenario. However, under a moderate climate and in the dystopian future (SoG) biodiversity loss is exacerbated in the Northern and Alpine regions by increasing forest removal. Conversely, the “water efficiency” strategy shows fewer, but more positive, impacts on other ecosystem services as, by enabling more cost-effective, continued irrigation, there is less need for large scale land use change. Therefore, positive relationships are seen in some regions with timber, atmospheric regulation and land use experience, and the negative biodiversity loss in the Northern and Alpine regions found in the other two strategies does not take place. There is, however, a reduction in arable biodiversity in the Northern region in the SoG scenario that is not observed under the “irrigation for food” strategy.

#### Adaptation strategies focussed on extensification and dietary change

Two of the adaptation strategies explore options for moving away from intensive agriculture. In the “extensify farming” adaptation strategy aims to produce a more natural landscape by reducing crop yields to reflect less intensive farming practices (yields are lower by choice as farmers are not maximising the potential of fields) and more land is put into set-aside. “Dietary change” follows the same extensive approach, but also modifies dietary preferences away from meat to reduce demand on the food system. Both adaptation strategies are run with food imports remaining at the socio-economic baseline level.

The modelling suggests that the “extensify farming” strategy shows a mixed response: extensive grassland/set-aside expands under WRW, but under SoG, where the pressure on the food system is great there is no notable increase. Furthermore, there is an increase in arable land cover and intensive grassland and a notable reduction in managed forests and unmanaged land in all scenarios (Fig. 5). Accepting lower yields means land is less productive, as such, more productive area (intensive grassland/arable) is needed to meet European food demand. This results inno notable change in food provision at the European level, but timber provision and atmospheric regulation decrease in many scenarios and regions, most notably in the Alpine, Atlantic and Continental regions where the expansion in arable is greatest. These declines are in general more extreme (more >20 % declines) and more common across regions and scenarios than in the “food self-sufficiency” adaptation strategy (Fig. 5).

Furthermore, the indicator of naturalness, “land use experience”, shows declines in many regions/scenarios, particularly in the Atlantic and Continental regions. This is because the increase in arable land set-aside for nature is more than counterbalanced by the loss of forest and unmanaged land. Arable biodiversity generally increases across regions and scenarios, particularly in Northern and Alpine regions, and forest biodiversity increases in the Southern region as a result of this adaptation strategy. However, there are declines >5 % in forest biodiversity in the Atlantic and Continental regions under SoG that are not present in the equivalent socio-economic scenario with the agriculture-centred “food self-sufficiency” strategy. Land use diversity improves in the Northern and Alpine regions in some scenarios as arable land use expands into new locations; however, it also declines in the Continental region in multiple scenarios as non-food producing land uses decline.

The “dietary change” adaptation strategy shows many of the same general trends, but impacts on the services of timber and atmospheric regulation are less severe, particularly in the extreme climate scenarios and the utopian scenario under moderate climate conditions. There are also fewer regional trends of declining land use experience >5 % than in the “extensify farming” strategy, and fewer trends >5 % loss of forest biodiversity, but more for arable biodiversity. These changes result from reductions in the demand for meat causing less land to be needed on which to rear animals, and less fodder crops are needed to feed them, reducing the demand for both intensive grassland and arable land. This is also reflected in the overall trend of declining food provision across scenarios and regions.

#### Adaptation strategies focused on forests and timber provision

Two of the adaptation strategies focus on the forestry sector. Both of these strategies include an adaptation option to increase food imports and so deliberately reduce pressure on the agricultural sector to allow land use change away from food-focused land uses. In addition, “maximising timber” aims to increase forest productivity by planting the most climatically suitable tree species for future conditions within existing forests, and “forests for nature” combines this with a doubling in protected areas, where new area is targeted at enlarging existing forests.

The two strategies show broadly similar results in terms of the land use change they encourage; more climatically suitable tree species allow forests in general to expand considerably (Fig. 5). In the dystopian scenarios, where, in the absence of adaptation, forests are a comparatively unprofitable resource (in comparison with the demand for food production land uses), the change in profitability that results from more appropriate planting leads to an increase in managed production forestry and an increase in timber provision (+89.3 to 101.0 Mt). As some of this new forest area comes at the expense of extensive grassland these changes lead to a reduction of the landscape experience index in both the “maximise timber” and the “forests for nature” adaptation strategies under SoG.

In the utopian scenario there is less demand for agricultural land and, hence, forestry is already quite competitive. Thus, in some cases the area of managed forest decreases, with forests changing to unmanaged. This increase in unmanaged forestry leads to significant increases in terms of the landscape experience index, but also to a decrease in timber production (>5 % decline at the European scale in WRW under both adaptation strategies: −24.7 to −28.0 Mt). In contrast to timber production, carbon sequestration shows positive trends in nearly all scenarios; this reflects the overall growth in total forestry.

Taking a regional perspective it is clear that the increase in forest growth is concentrated in the Continental and Southern regions which show increases in timber provision (>+20 %) in all four climate and socio-economic scenarios with both adaptation strategies. Furthermore in many scenarios, managed forestry moves out of the Northern, Alpine and Atlantic regions and into the Southern and Continental regions. These changes range from +38.8 Mt/year to −102.9 Mt/year with a scenario average of −14 Mt/year across the Northern, Alpine and Atlantic regions compared to scenario average gains of +33.6 Mt/year and +58.8 Mt/year for the Southern and Continental regions, respectively. This redistribution reflects the profitability of forestry in comparison with agriculture in the regions in question. As agriculture in the Southern region becomes more expensive/less competitive with climate change, forestry becomes a more viable alternative, particularly when climate appropriate species are planted.

In addition to these general similarities there are some notable differences between the two forest-oriented adaptation strategies. The “forests for nature” strategy sequesters more additional carbon under the dystopian scenarios than the “maximise timber” strategy (an additional +384/+1,135 Mt carbon per year in SoG under moderate and extreme climates, respectively). Conversely, “maximise timber” reduces carbon sequestration relative to the baseline in SoG combined with the moderate climate (−59.8 Mt/year) and only increases sequestration by 397.2 Mt/year when combined with the extreme scenario. This is because under the dystopian scenarios the expansion of protected areas restricts the use of land that would otherwise be put to “more profitable” uses, whereas in the utopian scenarios protected areas are not needed to encourage the expansion of forests, particularly unmanaged forests. In fact, buffering existing areas increases the amount of forestry already present and reduces the competitive advantage of new forestry.

#### Adaptation strategy focused on naturalness

The final adaptation strategy “go nature go!” aims to do whatever it can to maximise non-provisioning land uses. In addition to increasing imports, agricultural yields are increased to maximum (so that the same food can be grown using less land), dietary preferences are changed away from land-intensive meat production and climate-appropriate forests are planted. Furthermore, protected areas are doubled and targeted at a mix of land uses (forests, extensive grassland and unmanaged land). These new protected areas are prioritised in areas where there is currently no protection (to increase landscape connectivity), before buffering existing areas.

“Go nature go!” is exceptional in that it leads to extreme land use change (Fig. 5) with massive increases in unmanaged forestry in all scenarios, even the dystopian SoG where unmanaged forest growth was limited in the forest-targeted adaptation strategies. This difference is largely driven by assuming climatically suitable forests are being grown and protected, whilst food production is also reduced in all areas due to the combination of increased imports, increased yields and considerable dietary change towards vegetarianism. The results show significant impacts on food production which decreases (>−5 %) in all scenarios. Even with this decrease, the level of food production remains over 12,500 kcal capita^−1^ day^−1^ at the European scale in all scenarios. However, at the regional scale, the Northern, Continental and particularly the Alpine region see considerable reductions in food provision (>−20 % in many scenarios). In the Alpine region this reduces food provision to only 6,600 kcal capita^−1^ day^−1^ in the WRW and moderate climate scenario. Under this scenario inter-regional sharing would be essential for ensuring food security.

The “go nature go!” strategy has notable positive impacts on other ecosystem services, notably land use experience, carbon sequestration and forest biodiversity, all of which increase in a large number of regions and scenarios. Of these, the land use experience index has the greatest response, increasing in all regions and scenarios. This increase in naturalness is almost entirely a result of an increase in unmanaged forestry, and other non-provisioning land uses (unmanaged land and particularly extensive grassland) reduce considerably as a result of the strategy.

However, the adaptation strategy does have some negative influences: land use diversity decreases at the European scale in three of the four combined climate and socio-economic scenarios as a result of the reduction in arable and intensive grassland. Similarly, there is a large decrease (>−20 %) in habitat for species dependant on arable habitats at both the European and regional scales in all regions, but particularly the Northern and Alpine regions. Although alternative habitats may also be suitable for these species it is notable that likely candidates (extensive grassland, unmanaged land) are also declining as a result of the expansion in forestry.

## Discussion

### CLIMSAVE IAP in the broader context

Although not the first example of an integrated assessment model that combines meta-models to explore the implications of combined socio-economic, climate change and adaptation (c.f. Holman et al. 2005), the CLIMSAVE IAP is the first to do so at a European scale (Harrison et al. 2013) and this paper represents the first attempt to explore ecosystem services using such a system. Whilst other studies of ecosystem service delivery at the European scale have been applied, these are generally focussed on ecosystem service mapping and as such are often static, tied to a single time period or scenario (Zulian et al. 2014) and often only for an individual service (Zulian et al. 2013; Paracchini et al. 2014).

The presented results are broadly in agreement with the recent IPCC review (Kovats et al. 2014), in that climate change is expected to increase water stress and decrease biodiversity across Europe, with damaging impacts on food and timber production and carbon sequestration in southern Europe, but some beneficial impacts in Northern and Alpine regions. In general, changes in ecosystem service delivery are experienced in bundles (Raudsepp-Hearne et al. 2010) associated with the major land use classes. For example, there is a decrease in forest area, which tends to result in decreased timber production, carbon sequestration, landscape experience and forest biodiversity; whilst the increase in agricultural area contributes to an increase in land use diversity, food production and agricultural biodiversity indices.

This study also broadly supports the findings of Schroter et al. (2005), who simulated ecosystem service delivery for 2020, 2050 and 2080. Both studies identified similar increases in water stress in southern Europe and more notable decreases in agriculture in southern and central Europe relative to northern Europe. However, Schroter et al. identify different overall trends in land use, highlighting significant declines in agriculture and increases in forestry. This reflects a fundamental difference in methodological approach. The Schroter et al. (2005) study is driven by expert judgement and modelling in consultation with stakeholders, which provides an in-built reality check, but leads to a dependence on the assumptions with respect to future land use that are fed into the system. Conversely, land allocation in the CLIMSAVE IAP is driven by the overall profitability of land use, taking into account the cross-sectoral interactions between quantitative models of urban growth, flooding, water supply and demand, and potential yields from forestry and agriculture. Furthermore, the CLIMSAVE IAP is designed to model adaptation, including aspects such as innovation and behavioural change. This has considerable benefits for exploring the role of human agency within a given socio-economic scenario.

### Implications for decision-makers

The analysis above highlights a number of key messages relevant to decision-makers planning the future of European ecosystem services in two key areas. Firstly, it provides insight into the key driving forces behind potential future spatial patterns of ecosystem services; and secondly, it highlights potential trade-offs both between services themselves and between the regions that supply them.

#### Driving forces behind regional differences

The analysis highlights key ‘take-home messages’ with respect to four driving forces that play important roles in determining the future patterns of ecosystem services: (i) future climate; (ii) food demand; (iii) the effectiveness of innovations; and (iv) societal adaptation responses.

##### Future climate

The modelling shows that in the absence of societal intervention, and driven by profitability, future climate change causes agricultural-bundled ecosystem services to spread north, at the expense of grassland and managed forests (and their associated services) in many scenarios. Furthermore, stress on the water sector is likely to increase even under moderate climate change as a result of both changing water availability and increased demand for irrigation. Climate, therefore, enforces regional patterns of strength and weakness in terms of ecosystem service provision that can only be partially modified by societal changes. In this context the Southern region is likely to find itself with decreasing options whilst opportunities in the Northern region increase—both situations bringing with them difficult decisions with respect to ecosystem service trade-offs (see “Trade-off” in section).

##### Food demand

Meeting the food needs of the European population will be a key driver in future land use patterns with significant impacts on ecosystem service delivery. In situations with high levels of European food demand (e.g. SoG or the Food self-sufficiency adaptation strategy), large scale land use change is shown to be needed to meet this demand in the most profitable manner. This leads to loss of ecosystem services related to forest ecosystems. Whilst profitability may not be the only factor driving land use changes in reality, using land at less than profit-optimum will require a greater spatial expansion in agriculture to meet this demand, further increasing pressure on other ecosystems. To avoid such land use change an increase in food imports is necessitated. However, increasing imports could lead to a net global loss of biodiversity and ecosystem services if food production in supplier countries expands into high-biodiversity areas to meet increasing European demand.

##### Innovations

Where successful, the technological innovations modelled here reduce the pressure on ecosystem services by reducing the need for large-scale land use change in order to feed the population. Improvements in irrigation efficiency and water savings in the “Irrigation for food” adaptation strategy, for example, reverse the increase in agricultural area found in the Northern and Alpine regions in the same strategy without these improvements (“Food self-sufficiency”). In general, technological improvements as part of an adaptation strategy lead to benefits for ecosystem services under both socio-economic scenarios and both moderate and extreme climate change. However, there are limits to innovation success, as in some scenarios it was not possible to balance food and water provision without leading to critical levels of water stress. Furthermore, there is great uncertainty over the extent to which we will be able to achieve the modelled levels of innovation success in practice, highlighting that dependence on technological change alone may not be sufficient to prevent negative impacts on ecosystem services in the future. It also stresses the importance of maintaining existing levels of technology, as the declines in innovation success seen in SoG lead to significant changes in ecosystem services provision relative to baseline.

##### Societal adaptation responses

The different socio-economic scenarios and adaptation strategies show considerably different configurations for spatial configuration of European ecosystem services. This highlights the potential for adaptation options to make dramatic changes to the future provision of ecosystem services in Europe (e.g. “Go nature go!”). However, in many scenarios, ecosystem service protection requires quite notable societal change (e.g. a considerable decrease in societal preference for meat, a doubling of existing protected area targeted specifically at forestry, etc.). The challenges in implementing such societal changes in practice will be considerable.

#### Trade-offs

The “Go nature go!” adaptation strategy demonstrates that large scale land use change is theoretically feasible even under extreme climate and dystopian socio-economic scenarios. This means that, within the general constraints of climate, there are choices to be made on how Europe balances ecosystem services associated with agriculture and forests. However, not all areas will have the same opportunities to balance ecosystem service provision and, depending on the scenario, the Southern region may be in a position where neither forests nor agriculture are sufficiently profitable. Conversely, Northern regions may find themselves needing to balance the new opportunities for profitable agriculture with traditional forestry-based infrastructure and associated cultural heritage. These kinds of decisions may be particularly pertinent given that, in many scenarios, food demand for Europe cannot be met without the Northern region increasing agricultural production.

The adaptation strategies also showed that set-aside can lead to an increase in pressure on the agricultural system. Whilst it increases the available area for biodiversity in agricultural contexts (with potential synergies for pollination/pest control), under many scenarios this leads to more land being needed for food provision, aiding some arable species at the expense of species dependant on forests and unmanaged land.

Overall, the adaptation strategies highlight that it is not always possible to balance ecosystem service delivery across multiple sectors. Adaptation options were found that have synergies between sectors (e.g. between food and water or the bundled forestry/agricultural ecosystem services), but these often led to trade-offs in other sectors or regions (both within Europe and beyond). Furthermore, rebound effects were identified where adaptation strategies driving improvements in a sector actually increased pressure on the ecosystem services that they were intended to protect (e.g. irrigation/water improvements increase the profitability of irrigation, which leads to more irrigation and raises WEI).Such feedbacks need to be identified so that appropriate legislation can be put in place to ensure that strategies meet their aims without unintended consequences.

### Further extensions

The work presented here could be extended to explore optimum combinations of adaptation options for delivering different bundles of ecosystem services. Additionally, an approach similar to that of Jager et al. (2014) might be used to explore the implications for service delivery of different policy archetypes (ecosystem-based solutions; market-based solutions; technology-based solutions or people-based solutions) and combinations of these archetypes. Also, implications of these changes for human well-being could be explored by integrating a metric for coping capacity (Dunford et al. 2014) to identify where ecosystem service losses and gains overlap with areas that have the available capital (financial, natural, social, human or manufactured) to be able to cope with the negative consequences and make the most of the positive ones. Finally, as a further extension, the IAP’s land use classes could be scored for their ability to supply ecosystem services following a similar method to Burkhard et al. (2012). This would allow the effects of climate and socio-economic change to be investigated for a broader spectrum of ecosystem services.

## Conclusion

This study provides an overview of the potential future impacts of both climate and socio-economic change on ecosystem service delivery in Europe. Furthermore, it explores the implications of adaptation options and identifies the extent to which different combinations of options (strategies) work under different scenarios. The overall message is clear: climate change will have a significant impact on future ecosystem service provision, particularly in terms of the provision of water. In addition, socio-economic changes will lead to shifting patterns of service provision with more dystopian societies tending towards agriculture-based economies in an attempt to ensure food provision. Adaptation strategies are shown to offer significant opportunities to decrease pressures on the future provision of services. However, some of these changes will necessitate trade-offs with decisions needing to be made as to whether to focus on services bundled around the agricultural sector (for example, food provision, land use diversity and arable biodiversity) or those connected with forestry (for example, timber production, atmospheric regulation, landscape experience and forest biodiversity). Others allow synergies (such as between food and water provision), but these synergies will need time and resources to ensure their effectiveness. Furthermore, whilst the majority of adaptation strategies are able to mitigate climate impacts across multiple scenarios there are often unavoidable trade-offs between regions. Hence, coordination of adaptation actions will be essential to ensure that all needs are met: a factor that will become increasingly pressing under dystopian futures where inter-regional cooperation breaks down.

## References

[CR1] Alcamo J, Flörke M, Märker M (2007). Future long-term changes in global water resources driven by socio-economic and climatic changes. Hydrol Sci.

[CR2] Audsley E, Trnka M, Sabaté S, Maspons J, Sanchez A, Sandars D, Balek J, Pearn K (2014). Interactively modelling land profitability to estimate European agricultural and forest land use under future scenarios of climate, socio-economics and adaptation. Clim Chang.

[CR3] Berry PM, Rounsevell MDA, Harrison PA, Audsley E (2006). Assessing the vulnerability of agricultural land use and species to climate change and the role of policy in facilitating adaptation. Environ Sci Policy.

[CR4] Burkhard B, Kroll F, Nedkov S, Müller F (2012). Mapping ecosystem service supply, demand and budgets. Ecol Indic.

[CR5] Dubrovsky M, Trnka M, Holman IP, Svobodova E, Harrison PA (2014). Developing a reduced-form ensemble of climate change scenarios for Europe and its application to selected impact indicators. ClimChange.

[CR6] Dunford R, Harrison PA, Jager J, Rounsevell MDA, Tinch R (2014). Exploring climate change vulnerability across sectors and scenarios using indicators of impacts and coping capacity. Clim Chang.

[CR7] Gramberger M, Zellmer K, Kok K, Metzger M (2014). Stakeholder integrated research (STIR): a new approach tested in climate change adaptation research. Clim Chang.

[CR8] Harrison PA, Holman IP, Cojocaru G, Kok K, Kontogianni A, Metzger MJ, Gramberger M (2013). Combining qualitative and quantitative understanding for exploring cross-sectoral climate change impacts, adaptation and vulnerability in Europe. Reg Environ Chang.

[CR9] Harrison PA, Dunford R, Savin C-M, Rounsevell MDA, Holman IP, Kebede AS, Stuch B (2014). Cross-sectoral impacts of climate change and socio-economic change for multiple European land- and water-based sectors. Clim Chang.

[CR10] Harrison PA, Holman IP, Berry PM (2014b) Assessing cross-sectoral climate change impacts, vulnerability and adaptation: An Introduction to the CLIMSAVE project. Clim Chang (accepted)

[CR11] Holman IP, Rounsevell MDA, Shackley S, Harrison PA, Nicholls RJ, Berry PM, Audsley E (2005). A regional, multi-sectoral and integrated assessment of the impacts of climate and socioeconomic change in the UK. Clim Chang.

[CR12] Holman IP, Harrison PA, Metzger M (2014). Cross-sectoral impacts of climate and socio-economic change in Scotland—implications for adaptation policy. Reg Environ Chang.

[CR13] Jager J, Rounsevell MDA, Harrison PA, Omann I, Dunford R, Kammerlander M, Pataki G (2014). Assessing policy robustness of climate change adaptation measures across sectors and scenarios. ClimChange.

[CR14] Kok K, Sendzimir J, Bärlund I, Flörke M, Gramberger M, Zellmer K, Stuch B, Holman IP (2014). European participatory scenario development: strengthening the link between stories and models. Clim Chang.

[CR15] Kovats RS, Valentini R, Bouwer LM, Georgopoulou E, Jacob D, Martin E, Rounsevell M, Soussana J-F (2014) Europe. In: Barros VR, Field CB, Dokken DJ, Mastrandrea MD, Mach KJ, Bilir TE, Chatterjee M, Ebi KL, Estrada YO, Genova RC, Girma B, Kissel ES, Levy AN, MacCracken S, Mastrandrea PR, White LL (eds) Climate change 2014: Impacts, adaptation, and vulnerability. Part B: regional aspects. Contribution of working group II to the fifth assessment report of the intergovernmental panel on climate change. Cambridge University Press, Cambridge, pp 1267–1326

[CR16] Metzger MJ, Bunce RGH, Jongman RHG, Mücher CA, Watkins JW (2005). A climatic stratification of the environment of Europe. Glob Ecol Biogeogr.

[CR17] Paracchini ML, Zulian G, Kopperoinen L, Maes J, Schagner JP, Termansen M, Zandersen M, Perez-Soba M, Scholefield PA, Bidoglio G (2014). Mapping cultural ecosystem services: a framework to assess the potential for outdoor recreation across the EU. Ecol Indic.

[CR18] Raudsepp-Hearne C, Peterson GD, Bennett EM (2010). Ecosystem service bundles for analyzing tradeoffs in diverse landscapes. PNAS.

[CR22] Schroter D, Cramer W, Leemans R, Prentice IC, Araujo M, Arnell N, Bondeau A, Bugmann H, Carter T, Gracia C, de la Vega-Leinert A, Erhard M, Ewert F, Glendining M, House J, Kankaanpaa S, Klein R, Lavorel S, Lindner M, Metzger M, Meyer J, Mitchell T, Reginster I, Rounsevell M, Sabate S, Sitch S, Smith B, Smith J, Smith P, Sykes M, Thonicke K, Thuiller W, Tuck G, Zaehle S, Zierl B (2005) Ecosystem service supply and vulnerability to global change in Europe. Sci 310(25):1333–1337 10.1126/science.1115233 16254151

[CR19] Wimmer F, Audsley E, Savin C-M, Malsy M, Dunford R, Harrison PA, Schaldach R, Flörke M (2014). Modelling the effects of cross-sectoral water allocation schemes in Europe. Clim Chang.

[CR20] Zulian G, Maes J, Paracchini ML (2013). Linking land cover data and crop yields for mapping and assessment of pollination services in Europe. Land.

[CR21] Zulian G, Polce C, Maes J (2014). ESTIMAP: a GIS-based model to map ecosystem services in the European Union. Ann di bot.

